# Tea Polyphenols Mitigate Radiation-Induced Ferroptosis and Intestinal Injury by Targeting the Nrf2/HO-1/GPX4 Signaling Pathway

**DOI:** 10.3390/antiox14050580

**Published:** 2025-05-11

**Authors:** Runtian Li, Lintao Li, Haiyang Wu, Hui Gan, Zhuona Wu, Ruolan Gu, Xiaoxia Zhu, Shuchen Liu, Zhiyun Meng, Guifang Dou

**Affiliations:** 1 Beijing Institute of Radiation Medicine, 27 Taiping Road, Beijing 100850, China; 2 School of Public Health, University of South China, Hengyang 421001, China

**Keywords:** radiation-induced intestinal injury, tea polyphenol, ferroptosis, HSP90, gut microbiota, metabolites

## Abstract

Radiation-induced intestinal injury (RIII) is a significant concern for cancer patients receiving radiation therapy, as it can lead to complications such as radiation enteropathy. Presently, there are limited options for preventing or treating RIII. Tea polyphenols (TP), found in tea, provide various health benefits, but their antiradiation mechanisms are not fully understood. C57BL/6 mice pre-treated with TP for five days showed a significant improvement in survival rates after being exposed to 10 Gy of ^60^Co radiation. In the same way, abdominal exposure to 15 Gy of ^60^Co radiation effectively mitigated radiation-induced colon shortening, damage to intestinal tissues, oxidative stress, the release of inflammatory factors, and disruptions in intestinal microbial balance. In addition, TP treatment lowered the elevation of reactive oxygen species (ROS), iron imbalance, mitochondrial damage, and ferroptosis in IEC-6 cells post-irradiation. Utilizing network pharmacology, molecular docking, and affinity testing, we identified that TP has the capability to target the Nrf2/HO-1/GPX4 signaling pathway, while EGCG, a principal constituent of TP, interacts with HSP90 and mitigates radiation-induced ferroptosis. These findings suggest that TP may serve as a promising therapeutic agent to alleviate radiation-induced intestinal injury (RII).

## 1. Introduction

Radiation-induced intestinal injury (RII) is a major concern for patients receiving radiotherapy, especially those with abdominal or pelvic cancers, due to the gastrointestinal tract’s heightened vulnerability to ionizing radiation (IR). The exact cause of RIII remains unclear, but research indicates that it is linked to epithelial damage, vascular issues, and imbalances in intestinal immunity and microbiota [1,2,3]. There is currently no standardized or effective clinical method for preventing or treating RIII. This susceptibility is attributed to the rapid turnover and proliferation rates of gastrointestinal cells [4]. The type of cell death caused by radiation, including apoptosis, necrosis, autophagy-dependent death, and ferroptosis, depends on the dose of radiation and environmental factors [5]. Presently, Amifostine is the only therapeutic agent that the FDA has approved for the mitigation of RII. However, its effectiveness is limited, and the risk of serious adverse effects restricts its widespread use [6]. Therefore, it is crucial to find new options for treating RII.

Unlike apoptosis, necrosis, and autophagy, ferroptosis is a unique form of programmed cell death that depends on iron [7]. Ferroptosis is primarily influenced by increased levels of ROS and intracellular iron, lipid peroxidation, and the depletion of GSH [8]. MDA serves as an indirect biomarker for lipid peroxidation damage induced by ROS. Glutathione peroxidase 4 (GPX4), a member of the glutathione peroxidase family, is crucial in mitigating ferroptosis. GPX4 uses glutathione to reduce lipid peroxides, thereby defending cells against the possibility of ferroptosis [9]. Furthermore, exposure to radiation results in the downregulation of GPX4, which subsequently induces ferroptosis and is associated with an increased release of ROS and inflammatory mediators within cells [10]. Additionally, observations in acute kidney injury (AKI) cases have shown that ferroptosis, involving proteins such as Heat Shock Protein 90 (HSP90), can lead to the degradation of GPX4 through the chaperone-mediated autophagy pathway [11]. However, the role and regulatory mechanisms of the HSP90/GPX4 pathway in RIII have not been extensively investigated.

The gut microbiota and intestinal epithelial cells engage in bidirectional communication to sustain a balanced intestinal environment. The balance of gut microbiota can be disrupted, and the intestinal mucosal barrier compromised, by oxidative stress from excessive iron. Studies show that radiation therapy greatly changes the makeup of the gut microbiota, which is strongly linked to RIII [12]. Ferroptosis leads to oxidative stress that importantly disrupts the intestinal barrier, enabling microbial translocation and worsening dysbiosis. Concurrently, detrimental gut bacteria worsen iron overload and lipid peroxidation through pathways mediated by their metabolites [13]. This deleterious cycle highlights the interconnectedness of ferroptosis, redox imbalance, and microbiota homeostasis in the progression of RIII.

Tea polyphenols (TP) are naturally occurring compounds found in tea that have attracted considerable interest because of their potential health advantages. Known for their significant antioxidant effects, catechins, theaflavins, and thearubigins are prominent compounds. It is suggested that these antioxidant features help in reducing oxidative stress and might lower the risk of different diseases, such as cancer and cardiovascular disorders [14]. However, the capacity of tea polyphenols to mitigate RIII induced by irradiation exposure, along with the associated regulatory molecular mechanisms, remains inadequately understood.

In this research, we found that TP treatment prolonged the lifespan of mice exposed to radiation and reduced IR-induced ferroptosis in IEC-6 cells. Additionally, it lowered the inflammatory factor levels in the jejunum of mice exposed to radiation and modulated the irradiation-induced dysbiosis of intestinal flora and associated metabolites. Furthermore, our research provides new evidence showing that EGCG, the main component of TP, affects the GPX4 by interacting with the target protein HSP90, which in turn impacts the Nrf2/HO-1/GPX4 signaling pathway and mitigates radiation-induced ferroptosis. These findings underscore the potential therapeutic role of TP in the management of RIII.

## 2. Materials and Methods

### 2.1. Cells and Cell Culture

The IEC-6 cell line (Servicebio, Wuhan, China) was maintained in DMEM (Gibco, SuZhou, China) supplemented with 10% FBS (Cytiva, Shanghai, China) and 1% Penicillin–Streptomycin at pH 7.4 (Gibco, SuZhou, China) in a 5% CO_2_ atmosphere at 37 °C. Subculturing was performed bidaily using 0.25% trypsin.

### 2.2. Animals

Sourced from Beijing Vital River Laboratory Animal Technology Co., Ltd. (Beijing, China), male C57BL/6 J SPF mice weighing 20–22 g were acclimatized for three days, followed by an 8 h fast before radiation. Ethical approval was granted by the Ethics Committee of the Beijing Institute of Radiation Medicine (no. IACUC-DWZX-2024-P626, date: 6 March 2024), adhering to animal welfare standards.

### 2.3. UPLC-Q-TOF/MS Conditions for Ingredient Identification in TP

A UPLC system (SYNAPT G2-MS, Waters Corporation, Shanghai, China) used a Waters Acquity UPLC HSS T3 column (100 mm × 2.1 mm, 1.8 μm) to separate compounds at 25 °C with a flow rate of 0.2 mL/min. The detailed descriptions of UPLC-MS settings are shown in the Supplementary Materials.

### 2.4. Cell Viability Assay and Radiation Source

Cultured in 96-well plates at 3.5 × 10^3^ cells/well, IEC-6 cells adhered for 12 h before undergoing radiation. This study, approved by the Ethics Committee of the Beijing Institute of Radiation Medicine, involved treating cells with TP across a 0 to 200 μg/mL range in six replicates. After incubation periods of 24 and 48 h, 10 μL CCK-8 was added to each well and incubated at 37 °C for 45–60 min, and absorbance was measured at 450 nm using a microplate reader.

### 2.5. Calcein AM Staining

Cultured in confocal dishes, IEC-6 cells, post-radiation, were treated with TP concentrations up to 250 μg/mL. Post-24 h incubation, cells stained with calcein AM (Servicebio, G1609, Wuhan, China) for 15 min exhibited green fluorescence, captured using fluorescence microscopy.

### 2.6. Oxidative Stress Assay

The concentrations of glutathione (GSH, NJJCBIO, catalog no. A006-1-1, Nanjing, China), lactate dehydrogenase (LDH, NJJCBIO, catalog no. A020-2-2, Nanjing, China), superoxide dismutase (SOD, NJJCBIO, catalog no. A001-3-2, Nanjing, China), and malondialdehyde (MDA, NJJCBIO, catalog no. A003-1-2, Nanjing, China) were determined in accordance with the protocols provided by the manufacturer. Cells subjected to irradiation at doses of 8 Gy and 12 Gy were subsequently treated with varying concentrations of TP. This methodological approach ensures accurate assessments of oxidative stress markers in cellular environments following exposure to therapeutic levels of radiation.

### 2.7. Transmission Electron Microscopy (TEM)

Ultrastructural changes in IEC-6 cells due to irradiation were analyzed using TEM. Cells treated with trypsin were fixed in 2.5% glutaraldehyde and then washed and fixed again in 1% osmium acid. After dehydration, cells were embedded, sectioned, and stained with uranium acetate and lead citrate for mitochondrial examination via electron microscopy.

### 2.8. Flow Cytometry for Detecting ROS and Fe^2+^

IEC-6 cells, at 3.5 × 10^5^ cells/well in 6-well plates, underwent overnight incubation and 24 h exposure to varying TP concentrations (0, 50, 100, 150 μg/mL). Post-treatment, cells were rinsed twice with PBS, incubated with DCFH-DA (Beyotime, S0033S, Shanghai, China) for 30 min, and filtered through 0.4 μm nylon. Flow cytometry assessed ROS levels and intracellular Fe^2^⁺ using FerroOrange (DOJINDO, F374, Mashiki, Japan) with a BD FACS Aria analyzer (Franklin Lakes, NJ, USA).

### 2.9. Immunofluorescence

IEC-6 cells, irradiated in sterile dishes, received either 50 μg/mL TP or no treatment. The procedure involved fixing the cells with 4% paraformaldehyde, staining them with DAPI, and washing them with PBS. Following this, incubation was carried out overnight at 4 °C with antibodies directed against GPX4 (Affinity, Liyang, China, DF6701, 1:200) or HSP90 (Affinity, Liyang, China, AF6126, 1:200), followed by secondary antibody and DIPI dye treatment. Imaging was conducted using a Leica TCS SP8 SR confocal microscope (Leica) from Munich, Germany.

### 2.10. Western Blotting

Protein levels of HSP90, GPX4, Nrf2, Keap1, and HO-1 were quantified in hippocampal extracts via Western blot using antibodies from Proteintech (Rosemont, IL, USA) and Affinity (1:1000). The detailed description of Western blotting settings is shown in the Supplementary Materials.

### 2.11. Co-Immunoprecipitation (Co-IP)

After overnight incubation in 10 cm dishes, IEC-6 cells were lysed with Servicebio’s IP lysis solution (G2038-100ML) to extract proteins. The extracts were incubated with a 1:100 dilution of HSP90 antibody at 4 °C overnight, followed by binding to Protein A/G Sepharose beads (Millipore, IP05, Burlington, MA, USA) for three hours. After five washes, the beads were prepared for Western blot with loading buffer.

### 2.12. Transfection

A stable HSP90 knockdown line was established using a lentiviral vector encoding HSP90 RNA interference (RNAi, sequence: GGAAGAGAAAGGUGAGAAATT) and a control RNAi (sequence: CAGAGTATGTGTCTCGCATGAAG), both developed by Jiangsu Genecefe Biotechnology Co., Ltd., Wuxi, China. IEC-6 cells, seeded at 5 × 10^5^ cells per well in 6-well plates, underwent transfection with HSP90 RNAi for 12 h, with knockdown efficiency evaluated via Western blot. The RNAi base sequences are shown in Table S1.

### 2.13. Molecular Docking

The chemical structure of EGCG was obtained from the PubChem database. Concurrently, the three-dimensional molecular structure of the HSP90, designated by the PDB identifier 7S9H, was sourced from the RCSB PDB repository. The detailed description of molecular docking is shown in the Supplementary Materials.

### 2.14. Biolayer Interference Analysis

Binding affinity of HSP90 was determined with an Octet BLI Discovery12.2 (Göttingen, Germany) following Zhao et al.’s protocol [15]. HSP90 (RPA823Mu01, Cloud Clone Corp., Wuhan, China) was diluted in PBS with 0.02% Tween and biotinylated using a 10 mM solution at 25 °C for 30 min. The detailed description of the biolayer interference analysis is shown in the Supplementary Materials.

### 2.15. Analysis of TP’s Active Components and Their Corresponding Targets

A PubMed search using the keyword Tea polyphenols led to the identification of active compounds listed in ‘Drug.txt’. The SMILES notation (show in Table S2 for these molecules was then retrieved from PubChem (https://pubchem.ncbi.nlm.nih.gov/, accessed on 15 December 2024). Target genes related to these active compounds were systematically determined via SwissTargetPrediction (http://swisstargetprediction.ch/, accessed on 15 December 2024).

Gene data linked to ‘radiation damage’ were sourced from GeneCards (http://www.genecards.org/, accessed on 15 December 2024) and OMIM (https://www.omim.org/, accessed on 15 December 2024) into ‘Disease.txt’. These, along with ‘Drug.txt’, were merged into ‘Drug_Disease.txt’ using R 4.5.0.

### 2.16. Construction of the ’Active Ingredient–Target’ Network and Enrichment Analyses

Intersecting drug–disease genes were analyzed using STRING (https://cn.string-db.org/, accessed on 15 December 2024) to build a PPI network. Cytoscape 3.7.2 and cytoNCA were then employed to map interactions and identify key genes. GO and KEGG enrichment of core targets was performed with Metascape (https://metascape.org/, accessed on 15 December 2024).

### 2.17. Animal Grouping and Irradiation

All experiments used a ^60^Co γ-radiation source with a dose rate of 92.88 R/min. Mice were intragastrically administered TP or saline. The corresponding doses of TP were administered as a drug according to The Chinese Dietary Guidelines (2022). The details are as follows: In adults weighing 70 kg, human-equivalent dose (HED, mg/kg) = Mouse dose (mg/kg) × (Mouse Km/Human Km) [Km: the correction factor is estimated by dividing the average body weight (kg) of species to its body surface area (m^2^), Human Km = 70, Mouse Km = 3]. According to the HED, we calculated the dose for a mouse, and the animal dose is 100–160 mg/kg. In the survival study, 50 male mice were randomly assigned to five groups, each containing 10 mice: total body irradiation (IR), total body irradiation with TP at 110 mg/kg (IRTL), 220 mg/kg (IRTM), 440 mg/kg (IRTH), and total body irradiation with 150 mg/kg Amifostine (IRA). For the intestinal injury study, 50 mice were randomly allocated into five groups, each containing 10 mice: control (Con), total abdominal irradiation (IR), total abdominal irradiation with TP at 220 mg/kg (IRTL) and 440 mg/kg (IRTH), and total abdominal irradiation with Amifostine (IRA). After five days of treatment, mice in the IR, IRTL, IRTH, and IRA groups received 15 Gy of ^60^Co γ-irradiation. Mice were euthanized 3.5 days post-irradiation for organ collection.

### 2.18. Hematoxylin–Eosin (HE) Staining and Immunohistochemistry (IHC)

The small intestines were washed with cold PBS, fixed in 4% paraformaldehyde, and embedded in paraffin. Sections (5 μm) were stained with HE or IHC. IHC started with antigen retrieval followed by overnight incubation with primary antibodies at 4 °C. Antibodies used included GPX4, HO-1, HSP90, Keap1, and Nrf2. After incubation, secondary antibodies conjugated to horseradish peroxidase were applied, and DAB staining (ZSGB, ZLI-9019) was performed.

### 2.19. Fecal Sample Collection and Analysis Through 16S rRNA Gene Sequencing

The fecal samples were preserved in liquid nitrogen and kept at −80 °C for 16S rRNA sequencing by Majorbio Bio-Pharm Technology Co., Ltd. in Shanghai, China. Microbial diversity and abundance were assessed using alpha and beta diversity metrics.

### 2.20. Non-Targeted Metabolomics Determination of Mouse Serum

Serum was isolated from mouse plasma by centrifuging at 3000 rpm for 15 min and stored at −80 °C. The untargeted metabolomics analysis was carried out by Majorbio Bio-Pharm Technology Co., Ltd. in Shanghai, China. Using LC-MS with an ACQUITY UPLC HSS T3 column (100 mm × 2.1 mm, 1.8 µm, Waters, Milford, MA, USA), metabolites were extracted and isolated within a UHPLC-Q Exactive system by Thermo Fisher Scientific (Waltham, MA, USA). Both positive and negative ion modes were used, with a mass range of 70–1050 *m*/*z*.

Untargeted metabolomics of serum followed the method by Sun et al [16]. Data were normalized using the sum method to address sample variations, then log-transformed for feature comparability. Sparse partial least-squares discriminant analysis (sPLS-DA) was conducted using the mixOmics package in R 4.5.0for multivariate analysis.

### 2.21. Measurement of Cytokine Levels

Mouse serum was tested by Luminex using a Milliplex™ MAP kit (Millipore, Billerica, MA, USA) to measure IL-1β, IL-6, and TNF-α levels. The standard curve of IL-1β, IL-6, and TNF-α factor is shown in Figure S1.

### 2.22. Statistical Analysis

Data are presented as mean ± SEM from ≥3 biological replicates. GraphPad Prism 8.0 was used for statistical analysis. One-way ANOVA assessed group differences, while Student’s *t*-test compared pairs. Kaplan–Meier was applied for survival analysis. Statistical significance was set at *p* < 0.05.

## 3. Results

### 3.1. Components Identifying in TP

The main components of TP were analyzed using UPLC-TOF, and their composition was compared with a database using MS exact mass, MS/MS fragmentation, and the literature criteria. All identified components exhibit a quality precision of less than 5 ppm when compared to their theoretical values. From TP, 24 components were identified (Figure S1). Table 1 presents the primary and secondary MS details along with the structures of the eight identified compounds.

### 3.2. TP Markedly Enhanced HIEC-6 Cell Proliferation and Mitigated Oxidative Damage Following IR Exposure

**Figure 1:**
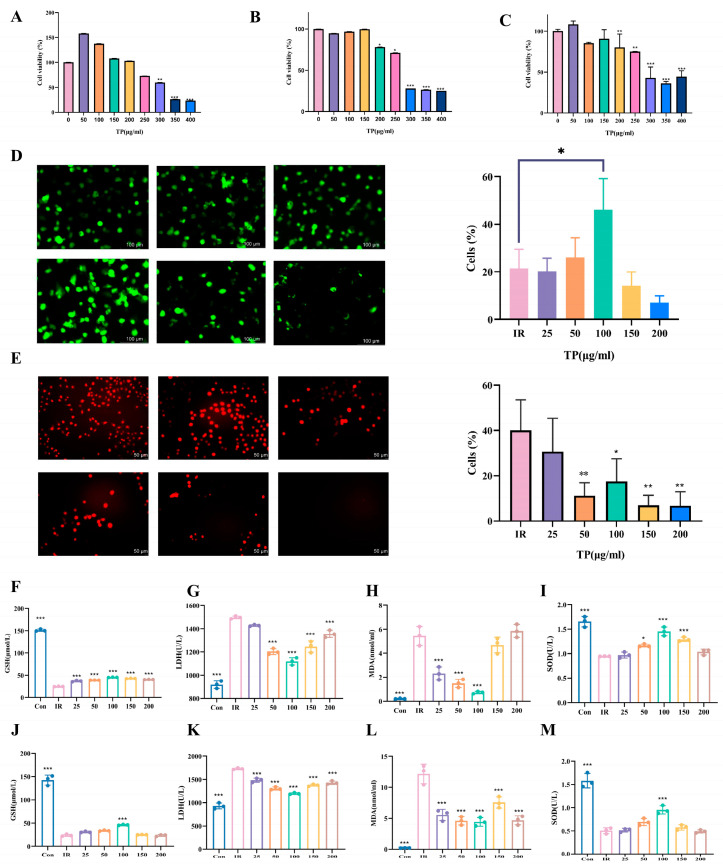
TP promoted IEC-6 cell proliferation after IR. (**A**–**C**) CCK-8 assays showed TP toxicity on IEC-6 cells at 24, 48, and 72 h (*n* = 6). (**D**) Calcein-AM stained with green representing live cell. Representative. Scale bar = 50 μm. The AM ratio was calculated. (**E**) The red representative fluorescent images of DCFH-DA in IEC-6 cells (*n* = 3). A quantitative analysis was performed. Cellular ROS was stained by red fluorescence. Scale bar = 100 μm. * *p* < 0.05, ** *p* < 0.01 vs. IR group. (**F**–**I**) GSH, LDH, MDA, and SOD levels in IEC-6 cells were measured 24 h after TP treatment post-radiation (*n* = 3, 8 Gy), * *p* < 0.05, *** *p* < 0.001 vs. IR group. (**J**–**M**) GSH, LDH, MDA, and SOD levels in IEC-6 cells were measured 24 h after TP treatment post-radiation (*n* = 3, 12 Gy), *** *p* < 0.001 vs. IR group.

The radioprotective mechanism of TP was studied in the IEC-6 cell line. Cell viability was assessed via CCK-8, revealing significant toxicity at ≥200 μg/mL, with survival rates > 50% at 72 h. Concentrations ≤ 50 μg/mL showed minimal toxicity (Figure 1A–C). Based on TP’s non-toxic radioprotective effects, 25, 50, 100, 150, and 200 μg/mL were selected for further experiments. Calcein-AM, a cell-permeable compound, was used to assess IR-induced cell death. TP at 150 μg/mL reduced IR-induced cell death (*p* < 0.05), as shown by calcein-AM staining (Figure 1D). ROS fluorescence detection showed a peak in ROS after radiation, which decreased upon TP treatment (*p* < 0.05). At TP concentrations above 25 μg/mL, ROS levels were markedly lower than in irradiated cells without TP (Figure 1E).

Previous studies highlight oxidative damage as a key factor in radiation injury [17]. After exposing cells to 8 Gy and 12 Gy doses, we assessed GSH and SOD levels (Figure 1F–M). A decrease in GSH was observed post-irradiation, but TP treatment elevated GSH or SOD levels, with the highest concentration of 100 μg/mL showing the most significant increase. The MDA and LDH levels, which rose following irradiation, were reduced by TP, with the greatest reduction also observed at 100 μg/mL.

### 3.3. TP Reversed the Irradiation-Induced Ferroptosis of ICE-6

**Figure 2:**
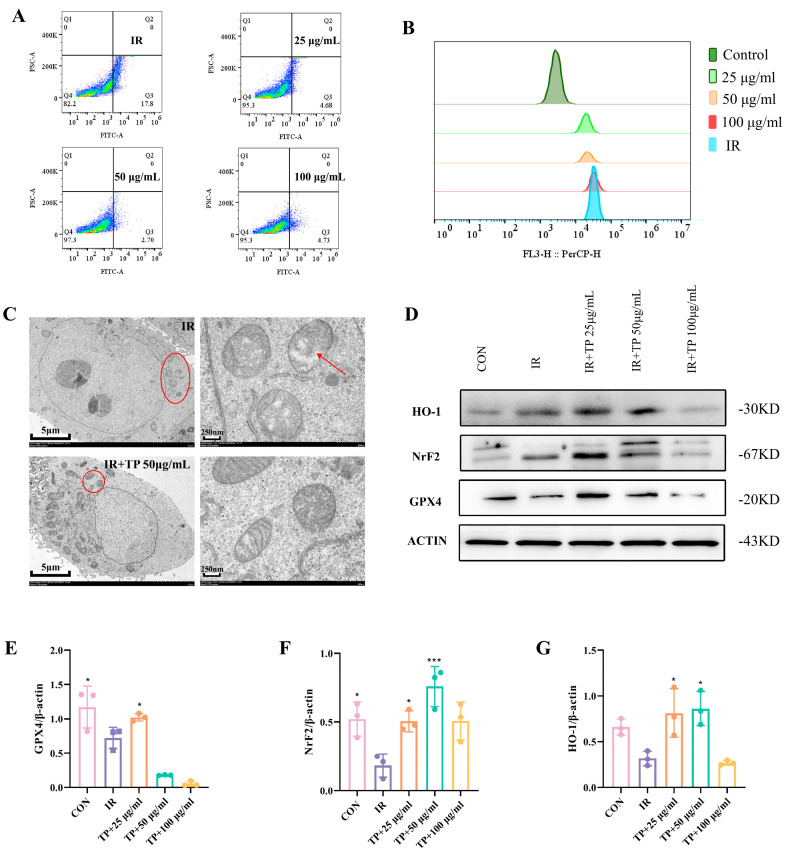
TP alleviated ferroptosis in IEC−6 cells post-irradiation. (**A**) The lipid ROS levels in IEC−6 cells were measured 24 h after TP treatment post-radiation and analyzed using a flow cytometer (*n* = 3). (**B**) The lipid Fe^2+^ levels in IEC−6 cells were measured 24 h after TP treatment post-radiation and analyzed using a flow cytometer (*n* = 3). (**C**) TEM imaging of irradiated cells was performed and measured 24 h after TP treatment post-radiation. Red circles indicate mitochondria, while red arrows highlight their enlarged structure. (**D**–**G**) Protein levels of HO−1, Nrf2, and GPX4 in IEC−6 cells were assayed by Western blotting, * *p* < 0.05, *** *p* < 0.001 vs. IR group.

Ferroptosis plays a central role in radiation-induced injury by promoting oxidative damage. Radiation exposure results in increased intracellular ROS and elevated intracellular Fe^2+^ concentrations, consequently inducing ferroptosis [18,19]. Given TP’s strong antioxidant properties, we used flow cytometry to confirm that 50 μg/mL TP effectively reduces ROS in ICE-6 cells exposed to radiation (Figure 2A). Following irradiation, Fe^2+^ levels in ICE-6 cells rose, but TP treatment at 50 μg/mL reduced Fe^2+^ levels, alleviating ferroptosis (Figure 2B). Mitochondria in ferroptotic cells shrink, with disrupted membranes and cristae [20]. Microscopy revealed radiation-induced mitochondrial damage, which TP at 50 μg/mL helped reverse by increasing mitochondrial number and maintaining structural integrity (Figure 2C). The Nrf2/HO-1/GPX4 signaling pathway was analyzed using Western blot to study the impact of TP on radiation-induced ferroptosis in IEC-6 cells. Compared to the control group, the expression of GPX4, HO-1, and Nrf2 was diminished following the irradiation of IEC-6 cells (*p* < 0.05). Conversely, in IEC-6 cells subjected to irradiation with TP treatment, the expression levels of these three genes were elevated relative to the IR group (Figure 2D–G).

### 3.4. Network Pharmacological Target Prediction

**Figure 3:**
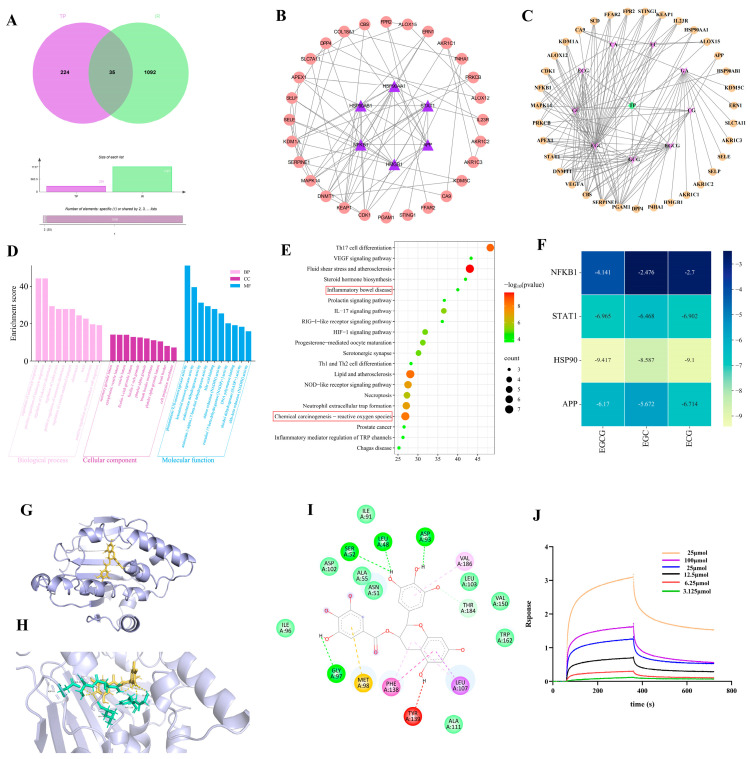
TP−IR network pharmacology analysis. (**A**) Venn diagram. (**B**) PPI network; purple represents the core target. (**C**) Drug–disease–target interaction. (**D**) GO enrichment analysis of TP for the treatment of RII. (**E**) Enrichment analysis of KEGG pathway for TP treatment of RII. (**F**) The heatmap illustrating the magnitude of binding energy of components in TP with respect to core target proteins. (**G**) Possible binding modes of EGCG to HSP90. (**H**) Structures of the binding pockets are shown by PyMOL 3.1 software. (**I**) Two-dimensional interactions of compounds and their targets. (**J**) BLI assay for EGCG and HSP90 binding affinity detection (K_d_ = 25 μmmol/L).

Using data from TCMSP and SwissTarget databases, 259 TP-related targets and 1127 RII targets were identified, with 35 common targets found (Figure 3A). We used the STRING database to create a network diagram of intersecting targets, imported it into Cytoscape, and excluded non-interacting targets to form the TP treatment RII protein–protein interaction network (PPI). The top-ranked targets, based on degree value, were identified as NFKB1, APP, STAT1, HSP90AB1, and HSP90AA1 (Figure 3B). The ‘drug–component–disease–target’ network suggested EGC, ECG, and EGCG as primary active compounds in TP’s effect on RII (Figure 3C). The primary biological processes identified through Gene Ontology (GO) analysis highlighted biological processes like leukocyte migration regulation, monocyte chemotaxis, and inflammatory response. Key molecular functions included DNA polymerase binding, nuclear receptor activity, and ketosteroid monooxygenase activity (Figure 3D). KEGG pathways identified reactive oxygen species, inflammatory bowel disease, IL-17 signaling, lipid metabolism, atherosclerosis, VEGF signaling, and necroptosis as pathways TP may influence in IR (Figure 3E). To assess the affinity of the components of TP for the core target, we performed molecular docking analyses. We calculated the binding energies of three components interacting with four proteins using Autodock Vina v.1.2.2. The results showed that EGCG had the lowest binding energy of -9.4 kcal/mol with HSP90, indicating a very stable interaction (Figure 3F). Molecular docking confirmed that EGCG directly interacts with HSP90, forming hydrogen bonds at Glya97, Sera52, Leua48, and Aspa93 (Figure 3G–I). Immediately after that, we performed an affinity assay of EGCG with HSP90 by BLI technique. The equilibrium binding of EGCG to HSP90 is concentration-dependent, with the strongest affinity observed at 25 μmol EGCG, as indicated by Rmax and Req values of 0.4765 and 0.629, respectively (Figure 3J).

### 3.5. TP Reduce Ferroptosis in IEC-6 Cells Post-Radiation by Targeting HSP90

**Figure 4:**
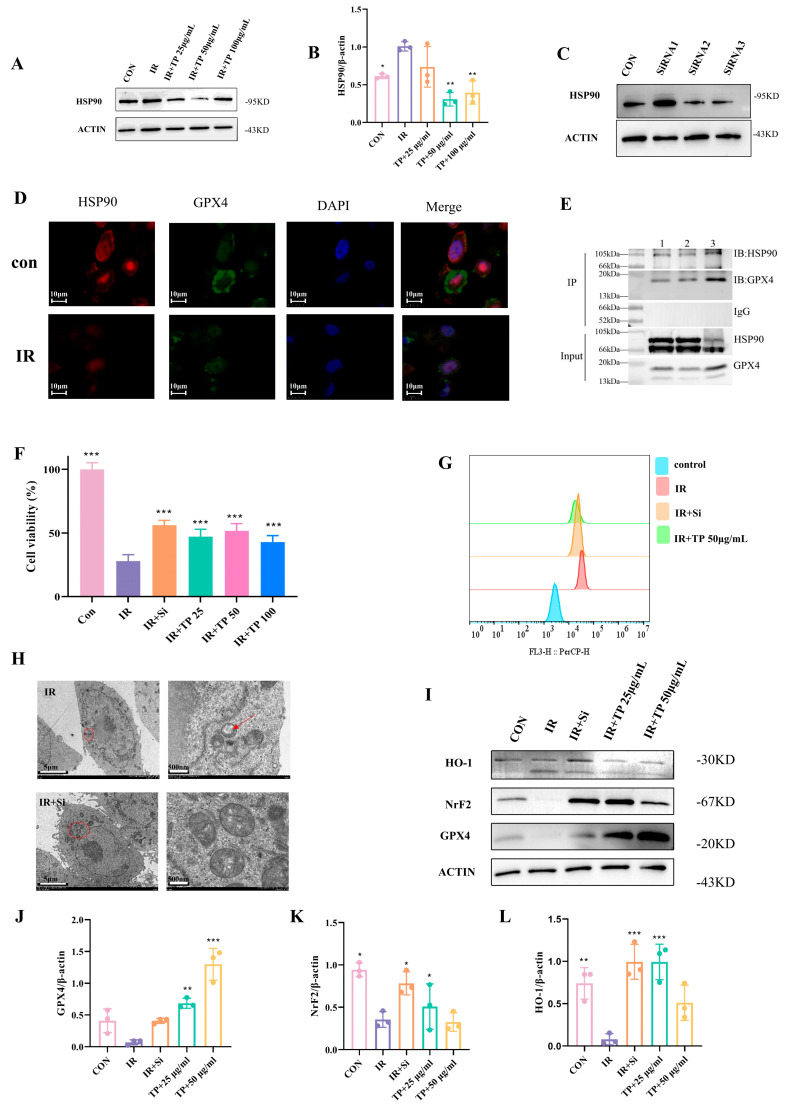
Mechanism of TP’s protection against radiation-induced damage. (**A**,**B**) WB analysis of HSP90 in IEC-6 cells after TP treatment, * *p* < 0.05, ** *p* < 0.01, vs. IR group. (**C**) WB of HSP90 in IEC−6 cells. (**D**) Cell viability was measured by CCK−8 in HSP90-siRNA transfected cells with TP treatment (*n* = 6), *** *p* < 0.001 vs. IR group. (**E**) Fe^2+^ levels in irradiated cells transfected with SiHSP90 and treated with TP were measured by flow cytometry. (**F**) Co−localization of HSP90 (red), GPX4 (green), and DAPI (blue). (**G**) Interaction between GPX4 and HSP90 was detected by immunoprecipitation. (**H**) TEM of irradiated IEC−6 cells: red circles indicate mitochondria, while red arrows highlight their enlarged structure. (**I**–**L**) HO−1, GPX4, and Nrf2 expression in irradiated cells (IR), SiHSP90-transfected cells (IR+Si), and cells treated with TP was analyzed by WB, * *p* < 0.05, ** *p* < 0.01, *** *p* < 0.001 vs. IR group.

Based on network pharmacological analysis, we proposed that HSP90 could be a key target for TP in reducing ferroptosis in IEC-6 cells. Subsequently, we conducted a WB assay to evaluate HSP90 expression levels. The findings indicated a significant upregulation of HSP90 expression in IEC-6 cells following irradiation, which was notably reduced upon treatment with TP (Figure 4A,B). Based on these observations, we hypothesized that the knockdown of HSP90 expression in IEC-6 cells could mitigate radiation-induced ferroptosis. Successful transfection of IEC-6 cells led to lowered HSP90 expression (Figure 4C). The CCK-8 assay assessed cell viability, demonstrating a considerable reduction in the survival rate of IEC-6 cells after they were irradiated. In contrast, the decrease was overturned in the HSP90 knockdown group, with IEC-6 cells treated with TP showing a higher survival rate compared to the irradiated group (Figure 4D). The HSP90 knockdown group had lower intracellular Fe^2+^ content than the irradiated group, as demonstrated by flow cytometry analysis (Figure 4E). In conclusion, we hypothesized that the HSP90 gene is implicated in the mechanism of ferroptosis caused by radiation in IEC-6 cells. We further co-localized the critical genes involved in ferroptosis, GPX4 and HSP90, within the cells using photofluorescence microscopy (Figure 4F). Co-immunoprecipitation (Co-IP) analysis corroborated the interaction between HSP90 and GPX4 and substantiated the significance of their mutual binding (Figure 4G). Simultaneously, transmission electron microscopy revealed that the mitochondrial morphology of IEC-6 cells exposed to irradiation remained intact following the knockdown of HSP90 (Figure 4H). We investigated the Nrf2/HO-1/GPX4 signaling pathway utilizing Western blot analysis and demonstrated that either the knockdown of HSP90 or the treatment of radiation-exposed IEC-6 cells with TP mitigated the radiation-induced alterations in the expression of all three genes, suggesting that HSP90 suppression decrease ferroptosis in IEC-6 cells (*p* < 0.05) (Figure 4I–L).

### 3.6. TP Alleviated Radiation-Induced Intestinal Damage in Mice

**Figure 5:**
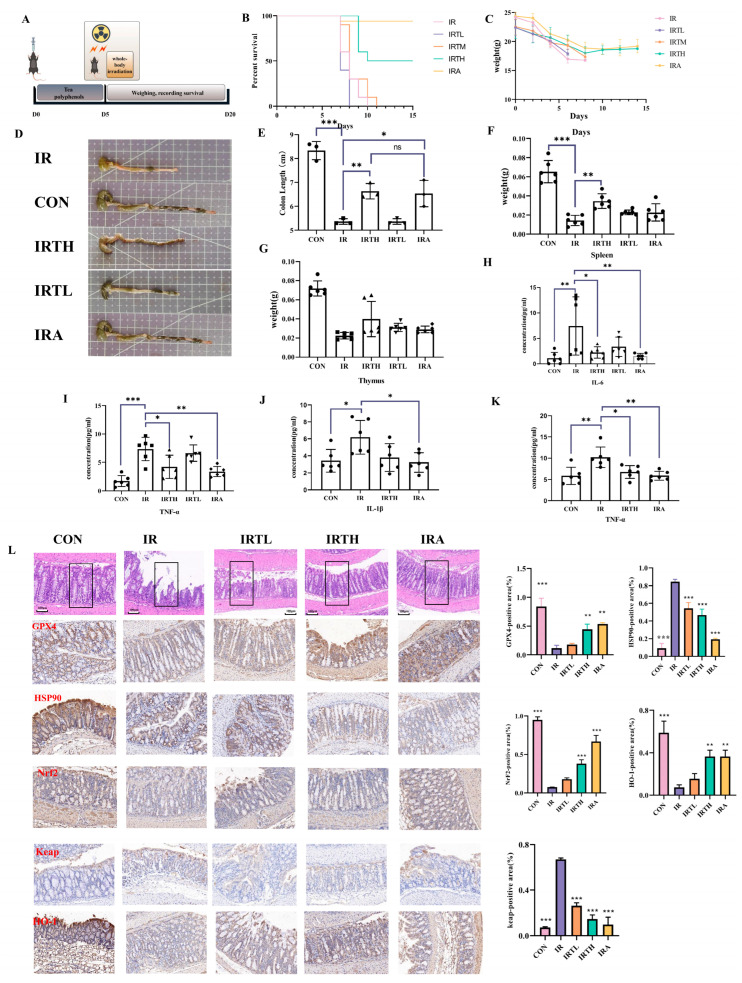
TP treatment alleviates radiation-induced damage in mice. (**A**) Experimental setup and treatment protocol. (**B**) Survival rates of mice post-radiation. (**C**) Average body weight post-radiation. (**D**,**E**) Colon length after radiation exposure (*n* = 6). (**F**) Average spleen weight after radiation (*n* = 6). (**G**) Average thymus weight post-radiation (*n* = 6). (**H**) Serum IL-6 levels (*n* = 6). (**I**) Serum TNF-α levels (*n* = 6). (**J**) Spleen IL-1β levels (*n* = 6). (**K**) Spleen TNF-α levels (*n* = 6). (**L**) HE staining of intestinal tissues post-radiation, GPX4, HSP90, keap, HO-1, NrF2 immunofluorescence in intestinal tissues post-radiation. * *p* < 0.05, ** *p* < 0.01, *** *p* < 0.001 vs. IR group.

TP was administered to C57 mice via intragastric delivery to assess their effects on radiation-induced intestinal injury (Figure 5A). In survival experiments involving systemically irradiated mice administered with TP treatment, it was observed that the IRTH group extended the survival duration of these mice (Figure 5B), while all groups experienced a consistent decline in body weight over time (Figure 5C). The effect of abdominal irradiation was a reduction in colon length, but the IRTH and IRA groups showed longer colons than the IR group (*p* < 0.05) (Figure 5D,E). As key immune organs, the thymus and spleen play vital roles in radiation protection. The spleen weight in the IRTH group exceeded that of the IR group (*p* < 0.05), and the thymus also showed increased weight (Figure 5F,G). In radiation-induced intestinal injury, higher levels of inflammatory factors like IL-1β, IL-6, TNF-α, and TGF-β correlate with the severity of damage. These factors increase intestinal permeability, cause neutrophil infiltration, damage the mucosal barrier, and trigger inflammation, worsening injury. Post-radiation serum levels of TNF-α and IL-6 were elevated compared to the treatment and control groups (*p* < 0.05) (Figure 5H,I). The investigation of inflammatory markers in the spleen showed that IL-6 levels were the same across all groups (show in Figure S3), whereas IL-1β and TNF-α were decreased in the IRTH, IRA, and control groups relative to the radiation group (*p* < 0.05) (Figure 5J,K). Pathological analysis of the intestine revealed substantial inflammation and disorganization of the mucosal glands in the IR group (Figure 5L). The immunohistochemical analysis showed a significant decrease in the levels of HO-1, Nrf2, keap, and GPX4 in enterocytes after exposure to radiation. The IRTP group also demonstrated a considerably larger positive staining area than the IR group. Conversely, the IR group had a higher level of HSP90 gene expression than any other group (*p* < 0.05) (Figure 5L).

**Figure 6:**
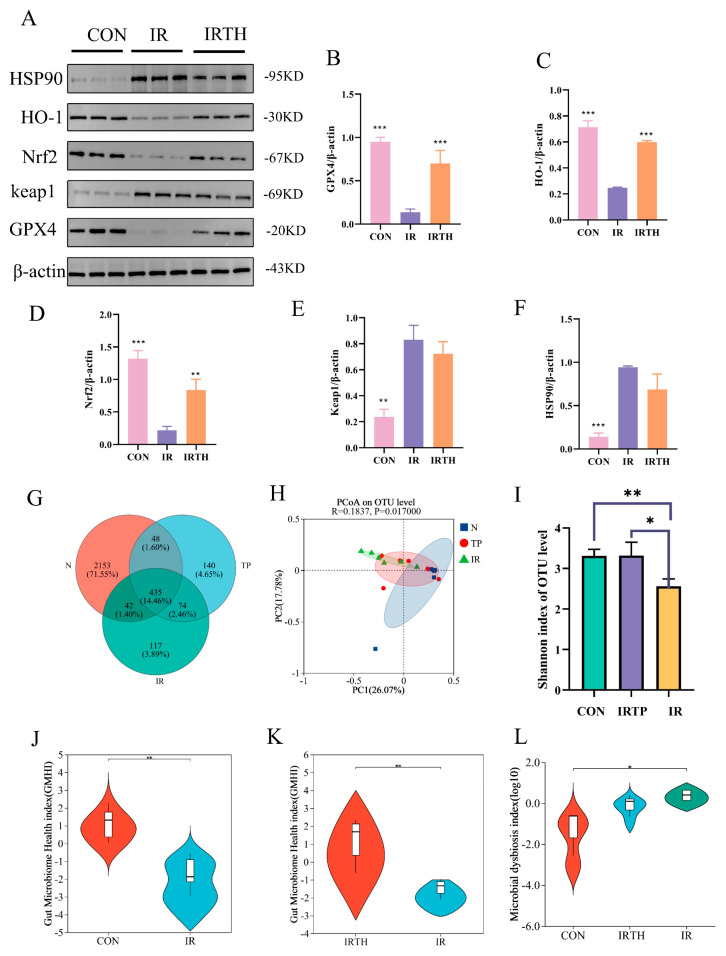
Gut microbiota diversity in IR mice. (**A**–**F**) Protein levels of HSP90, HO−1, Nrf2, keap1, and GPX4 in intestine were assayed by Western blotting. (**G**) Venn diagram. (**H**) PCoA plot. (**I**) Shannon index (*n* = 6). (**J**–**L**) GMHI and MDI analysis (*n* = 6). * *p* < 0.05, ** *p* < 0.01, *** *p* < 0.001 vs. IR group.

Radiation triggers oxidative stress and inflammation in the small intestine by elevating ROS levels, ultimately resulting in ferroptosis. This study explored the effects of TP on oxidative stress signaling pathways within the small intestine tissues of irradiated mice using WB analysis and examined the expression of related genes. The results showed that TP treatment reduced radiation-induced GPX4 expression and improved RII by influencing the Nrf2/HO-1/Keap1 signaling pathway (Figure 6A–F).

### 3.7. Effects of TP on Gut Microbiota in Radiation-Exposed Mice

A Venn diagram (Figure 6G) was used to analyze OTU/ASV values, reflecting microbial diversity across groups. Post-IR, mice showed reduced gut microbiota, which was partly restored by TP. Principal Coordinates Analysis (PCoA, Figure 6H) revealed significant differences between the IR and CON groups, with TP treatment partially mitigating this effect. The Shannon index (Figure 6I), representing α diversity, was lower in the IR group (*p* < 0.05), indicating reduced microbial richness, but TP did not effectively restore these indices. The coverage index was approximately 1 for all groups, indicating sufficient sequencing depth to cover most microorganisms, including rare species. The Gut Microbiome Health Index (GMHI), a predictor of gut health, was lower in the IR group (Figure 6J,K) and improved with TP treatment, suggesting that TP could restore gut microbiota balance. The Microbial Dysbiosis Index (MDI, Figure 6L) was higher in the IR group (*p* < 0.05), indicating dysbiosis, with no notable difference between CON and IRTH groups.

### 3.8. Gut Microbiota Community Analysis

**Figure 7:**
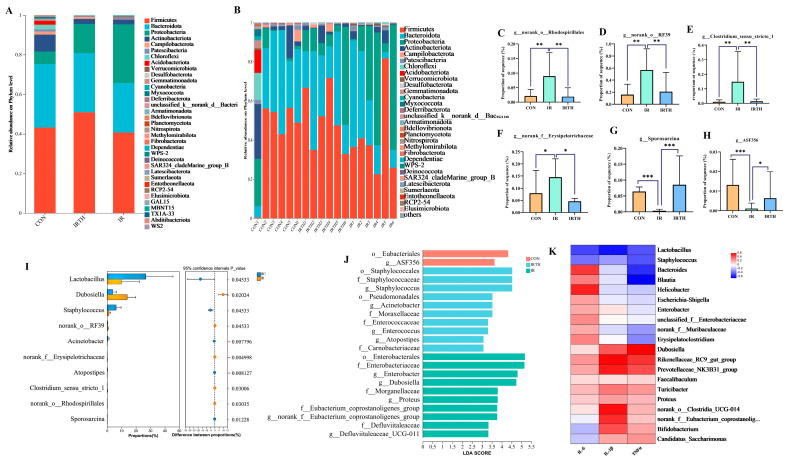
Gut microbiota analysis. (**A**,**B**) Phylum-level comparisons. (**C**–**H**) Kruskal−Wallis test for identifying changes in abundance of *Erysipelotrichaceae*, *Clostridium_sensu_stricto_1*, *Rhodospirillales, RF39, Sporosarcina*, and *ASF356*. *n* = 6, * *p* < 0.05, ** *p* < 0.01, *** *p* < 0.001 vs. IR group. (**I**) Abundance differences between IR and TP groups. (**J**) LEfSe analysis of group differences. (**K**) Correlation between microbiota and inflammatory factors (top 20 genera).

Microbial composition was analyzed at both the genus and species levels in the three mouse groups. At the genus level, the IR group showed a reduction in beneficial probiotics, including *norank_f__Muribaculaceae*, *Lactobacillus*, and *Bifidobacterium*, with an increase in pathogenic bacteria such as *Escherichia-Shigella* and *Enterobacter*. These changes were reversed with TP administration, as shown in Figure 7A,B. A species-level analysis in Figure 7B revealed differences in the dominant species and their proportions among the groups. Using the Kruskal–Wallis test to analyze intergroup variations, we found a rise in the abundance of harmful bacteria such as *g__norank_f__Erysipelotrichaceae*, *g__Clostridium_sensu_stricto_1*, *g__norank_o__Rhodospirillales*, and *g__norank_o__RF39* (*p* < 0.05) together with a decline in advantageous bacteria like *g__Sporosarcin*a and *g__ASF356* (*p* < 0.05) (Figure 7C–H). Group comparisons (Figure 7I) highlighted that *Lactobacillus* (*p* < 0.05) was more abundant in the TP group compared to the IR group. These findings indicate that the gut microbiota composition, species dominance, and abundance varied across the groups, potentially influencing disease progression and the pharmacological effects of TP.

LDA analysis of gut microbiota across the three groups identified key biomarkers with varying levels of abundance (Figure 7J). A total of 224 and 14 microbial groups were found in the CON, IR, and IRTH groups, respectively. The CON group showed significant bacteria, including *o__Eubacteriales* (LDA = 4.32) and *g__ASF356* (LDA = 3.63). In the TP group, the most significant bacteria were *f__Staphylococcaceae* (LDA = 4.52), *g__Staphylococcus* (LDA = 4.52), and *o__Staphylococcales* (LDA = 4.52). The IR group was characterized by significant bacteria such as *o__Enterobacterales* (LDA = 5.16), *f__Enterobacteriaceae* (LDA = 5.14), and *g__Enterobacter* (LDA = 4.80). A microbial interaction network was also constructed, revealing a synergistic relationship among *g__norank_f__JG30-KF-CM45*, *g__norank_f__Vicinamibacteraceae*, and *g__Arthrobacter*. The correlation heatmap showed negative correlations between IL-1β and *Lactobacillus* and between TNF-α and *Blautia* in the IRTH/IR comparison (Figure 7K), consistent with studies indicating these bacteria reduce inflammation.

### 3.9. Serum UPLC-MS Untargeted Metabolomics Analysis

**Figure 8:**
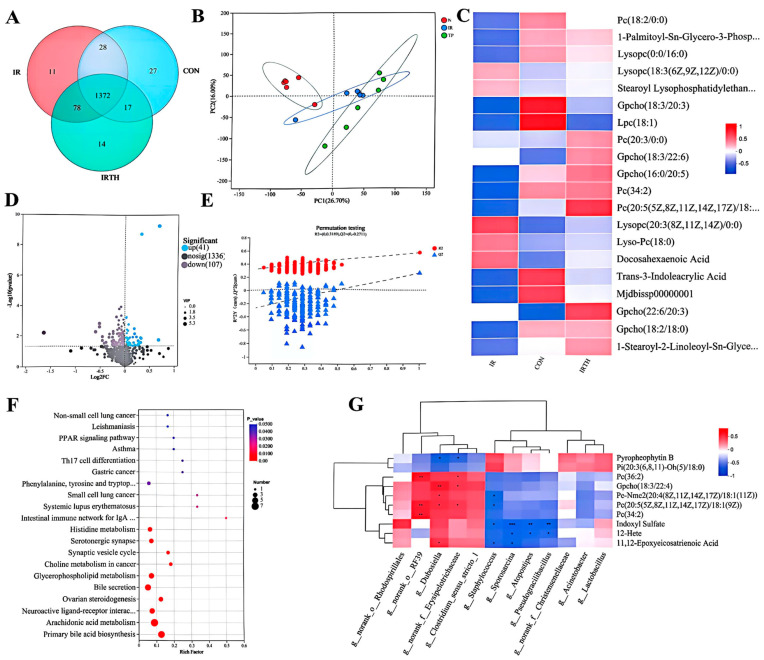
HPLC−MS untargeted metabolomics. (**A**) Venn diagram. (**B**) PCA analysis for all groups in both ion modes. (**C**) Heatmap of top 20 differential metabolites. (**D**) PLS−DA models with displacement test results. (**E**) Volcano plot: IR vs. IRTH groups. (**F**) Pathway analysis of differential metabolites. (**G**) Correlation of metabolites and microbiota at phylum/genus levels (top 20). Red: upregulated, blue: downregulated. Significant positive and negative correlations are indicated by red and blue squares, respectively. * *p* < 0.05, ** *p* < 0.01, *** *p* < 0.001.

Three distinct serum metabolite groups were identified, with 1372 metabolites shared across all groups (Figure 8A). PCA of the serum samples in both cation and anion modes (Figure 8B) showed clear differentiation among the groups, reflecting significant heterogeneity. A heatmap illustrating the top 20 differential metabolites across the three groups is presented (Figure 8C). The QC samples clustered tightly, verifying experimental stability. PLS-DA was applied to explore the relationship between metabolite expression and groupings (Figure 8D). A permutation test with 200 iterations confirmed the model’s robustness, as shown by the lower Q2 and R2 values on the left side and the intersection of the Q2 regression line with the Y-axis below zero, validating the model’s reliability. Differential metabolites were identified based on criteria of Fold Change > 1, VIP ≥ 1, and *p* < 0.05. Volcano plots (Figure 8E) identified 148 metabolites in the TP group compared to the control, with 41 upregulated and 107 downregulated.

TP intervention affected key pathways such as IgA production, Th17 cell differentiation, and bile acid biosynthesis (Figure 8F). Phosphatidylcholine (PC) metabolites positively correlated with *g__norank__o__RF39* (Figure 8G), a genus that may alleviate DSS-induced inflammation [21].

## 4. Discussion

Ionizing radiation presents various health risks, particularly in its expanding medical and industrial use, causing direct cellular damage and harmful biochemical reactions affecting multiple body systems [22]. Amifostine is a radioprotective agent used in cancer treatments to protect cells and combat radiation-induced hematopoietic damage. However, its intravenous administration and side effects like hypotension, nausea, vomiting, and allergic reactions limit its clinical use [23]. Traditional Chinese Medicine (TCM) and its compounds offer a promising approach to radiation protection, boasting multicomponent, multilevel, multipathway, and multitarget therapeutic effects. Compared to other antiradiation products, TCM is effective, low in toxicity, cost-efficient, and highly efficient, making it a strong candidate for radiation protection.

Ferroptosis is a recently discovered type of programmed cell death that differs from conventional apoptosis, marked by its dependence on lipid peroxidation and iron buildup [24]. Research has demonstrated that ionizing radiation may initiate cell death through iron induction [25]. This process is influenced by lipid peroxidation, where ROS causes oxidative damage to lipids, ultimately leading to cellular harm and membrane instability. Lipid peroxidation, ROS, and iron metabolism interact in a complex way, where they reinforce each other; ROS aids in iron accumulation, and iron boosts ROS, leading to increased oxidative stress. High concentrations of iron and lipid peroxidation ROS can cause considerable harm to cell membranes [26]. Mitochondrial structural and functional changes are central to ferroptosis, marked by smaller mitochondrial volume, denser membranes, fewer mitochondria, and reduced mitochondrial membrane. Furthermore, we observed an accumulation of iron ions in IEC-6 cells following irradiation, suggesting that radiation induces ferroptosis. Our observations of mitochondrial structure revealed that radiation exposure resulted in a reduction of mitochondria and disruption of mitochondrial integrity in IEC-6 cells. Additionally, the iron death-related gene GPX4 was downregulated after irradiation, as determined by Western blot analysis, further corroborating that irradiation exposure induces ferroptosis in cells. Therefore, targeted modulation of radiation-induced cellular iron death has emerged as a promising therapeutic strategy. Previous research on antiradiation drugs has predominantly concentrated on their antiapoptotic [27] and anti-inflammatory properties [28]. However, there is a notable deficiency in research and development concerning antiradical drugs that exhibit resistance to radiation-induced ferroptosis. Furthermore, the cellular mechanisms triggered by radiation remain inadequately understood, underscoring the innovative potential of investigating TP in the context of antiradiation ferroptosis. This study aimed to evaluate whether TP mitigates irradiation-induced ferroptosis in IEC-6 cells and to elucidate the underlying mechanisms. Lipid peroxidation and iron accumulation levels in IEC-6 cells were assessed after TP intervention using FerroOrange and DCFH-DA fluorescent probes, in addition to GSH, LDH, SOD, and MDA assay kits. Additionally, Western blot analysis was employed to examine the expression of key targets involved in iron oxidation, including GPX4. We found that TP diminished Fe^2+^ levels, promoted GSH accumulation, curtailed intracellular lipid ROS and MDA buildup, mitigated damage to mitochondrial structures, and upregulated GPX4 expression in irradiation-exposed IEC-6 cells, thereby attenuating the radiation-induced ferroptosis response.

Recent research has indicated a link between Nrf2/HO-1 and ferroptosis, with studies also showing a close connection to radiation-induced lung injury [29]. In response to xenobiotic and oxidative stress, the Nrf2 signaling pathway is vital for reducing toxic impacts and safeguarding organs like the kidneys, liver, nervous system, and lungs [30,31,32]. Our comprehensive enrichment analysis, utilizing network pharmacology, elucidated the principal mechanisms by which TP treatment ameliorates radiation damage, suggesting that TP may activate the oxidative stress signaling pathway associated with reactive oxygen species to confer antiradiation effects. Therefore, the HO-1/Nrf2 signaling pathway was analyzed through WB, indicating that its upregulation is inversely associated with ferroptosis, confirming the results of Shan et al. [33].

As a vital molecular chaperone, HSP90 is involved in cellular signaling and stress response, helping client proteins mature and become active. In both healthy and unhealthy cellular functions, HSP90 is essential. It manages programmed cell death mechanisms like apoptosis, autophagy, necroptosis, and ferroptosis in tissues that are not functioning properly [34]. Numerous studies indicate that HSP90 could be a potential target in ferroptosis, but its exact function is still debated [35]. The study by Su et al. revealed that GA, an HSP90 inhibitor, depletes GSH and accelerates ferroptosis, but another HSP90 inhibitor effectively reduces ferroptosis in cardiomyocytes [36]. This study employed network pharmacology to identify the primary core target of TP in treating radiation injury for the first time. Through molecular docking analysis, we determined that the binding energy of EGCG within TP to HSP90 was a minimum of −9.4174 kcal/mol. Subsequent affinity testing confirmed a strong binding interaction between EGCG and HSP90. Zhang et al. demonstrated that the suppression of HSP90 expression led to a significant upregulation of GPX4, thereby mitigating ferroptosis [37]. Analysis of HSP90 expression in IEC-6 cells revealed a significant upregulation following irradiation exposure. Further experiments involving HSP90 knockdown via transfection prior to irradiation demonstrated a notable increase in cell survival and a reduction in intracellular ferric ion concentration, suggesting a potential link between HSP90 and ferroptosis. Co-immunoprecipitation assays revealed an interaction between HSP90 and GPX4. Additionally, examination of GPX4 expression in IEC-6 cells with HSP90 knockdown before irradiation indicated a significant upregulation of GPX4. In summary, we found that HSP90 is likely to be a potential target for radiation-induced cellular iron death and that down-regulation of HSP90 reduces radiation-induced cellular ferroptosis. Research indicates that activating HO-1 by inhibiting HSP90 can lessen infarct size, fibrosis, and macrophage infiltration in a model of myocardial ischemia/reperfusion [38]. This study also demonstrated that the HO-1/Nrf2/GPX4 signaling pathway was activated in IEC-6 cells with HSP90 knockdown.

In our prior investigation, we administered TP at three different concentrations: low, medium, and high doses. The outcomes revealed that the high-dose group prolonged the mice’s lifespan and lessened the intestinal damage due to radiation. Moreover, this high-dose treatment provided effective protection for intestinal cells and minimized inflammation-related damage. Compared to the irradiated mice, the group receiving TP intervention showed notably lower levels of HSP90 and GPX4 expression in the intestines. Additionally, tea polyphenols, particularly EGCG found in green tea, have been shown to effectively reduce intestinal inflammation and injury through the regulation of gut microbiota and enhancement of intestinal barrier function [39].

The histopathological analysis of HE stained sections of the small intestine in this study revealed that irradiation led to disruption, shortening, and inflammatory infiltration of the intestinal villi in C57 mice. Furthermore, subsequent quantification of inflammatory markers in both serum and spleen indicated an elevation of these factors in the irradiated group compared to controls. Ionizing radiation is extensively documented for its role in elevating inflammatory markers by disrupting cellular processes and triggering immune responses. It induces inflammation in murine macrophages, leading to increased expression of genes associated with inflammation, and contributes to systemic inflammation through the release of cytokines [40,41]. This study employed network pharmacology to investigate the protective mechanisms of TP against radiation-induced damage, with KEGG pathway analysis revealing the activation of inflammatory signaling pathways. Inflammatory markers in the serum and spleen of irradiated mice were assessed, showing an increase in IL-1, IL-6, and TNF-alpha levels following radiation exposure. Notably, TP treatment suppressed the expression of these inflammatory cytokines (*p* < 0.05).

Our investigation revealed that TP treatment not only improved survival rates and facilitated the recovery of intestinal injury but also played a crucial role in restoring the balance of gut microbiota. It notably increased the abundance of beneficial probiotics in the gut. A healthy gut microbiota is characterized by high microbial richness, diversity, and a stable composition. Numerous studies have shown that radiation exposure leads to profound alterations in gut microbiota, which is closely associated with radiation-induced intestinal damage [42]. In our study, radiation exposure caused a significant reduction in the diversity and richness of the gut microbiota, as evidenced by a decrease in the Shannon index. Additionally, the GMHI was considerably lower in the radiation-exposed group compared to the healthy controls, suggesting a deterioration in gut health following radiation exposure. These negative effects were notably lessened by the administration of TP. The role of Firmicutes in gut health has gained increasing recognition as the microbiome has been studied more thoroughly. Higher levels of Firmicutes are often associated with better metabolic health and enhanced immune function. These bacteria aid host metabolism by enhancing the creation of short-chain fatty acids (SCFAs), which play a role in controlling inflammation and fortifying the intestinal barrier [43]. Our findings show that TP treatment increased the abundance of Firmicutes in the gut microbiota of irradiated mice, which likely contributed to the observed anti-inflammatory effects. Actinobacteriota, and in particular bifidobacteria, are essential for the proper functioning of the immune system. These bacteria stimulate the production of mucosal immunoglobulins, promote intestinal lymphocyte activation, and foster a balanced immune response [44]. Following radiation exposure, the levels of Actinobacteriota in mice were reduced. Compared to the irradiated group, the TP-treated group demonstrated a significant boost in Lactobacillus levels. The reduction of pro-inflammatory cytokines, including TNF-α, by Lactobacillus strains contributes to mitigating inflammation and alleviating related diseases. Consistent with this, Lactobacillus supplementation in mice lowered TNF-α levels, indicating its protective role in modulating the immune response [45].

The UPLC-MS untargeted metabolomics analysis revealed that both radiation exposure and TP administration affect various metabolic pathways, including those involved in retinol metabolism, phosphonate and phosphinate metabolism, vitamin B6 metabolism, lipoic acid metabolism, and the biosynthesis of primary bile acids. These pathways are crucial for energy metabolism and immune function. Notably, retinol metabolism has attracted significant attention because of its essential role in sustaining health. As a fat-soluble vitamin, retinol is indispensable for cellular growth, differentiation, and the functioning of the immune system. Interestingly, low levels of retinol in the serum are often associated with chronic inflammation, a hallmark of several metabolic disorders [46]. TP treatment also promotes the differentiation of Th17 cells and enhances the intestinal immune network crucial for IgA production, which is important for maintaining immune stability. IgA is the most abundant immunoglobulin in mammals and is primarily synthesized in the gut. Its production depends on both T cell-dependent and-independent pathways and is influenced by the gut microbiota. New research indicates that the makeup and variety of gut microbiota play a crucial role in influencing IgA production, which is vital for preserving immune equilibrium in the gastrointestinal tract [47].

Several constraints are present in this study. Firstly, the antioxidant mechanism by which TP modulates RII is not yet fully elucidated, highlighting the need for more validation. Secondly, this study’s lack of focus on the bioavailability, metabolic differences, and long-term safety of TP in humans reduces its potential for clinical application. Thirdly, our in vitro research was limited to IEC-6 cells, a single cell line. Future studies will aim to investigate multiple cell lines and employ organ-like models to more accurately simulate the in vivo intestinal environment and explore the specific mechanisms involved. Finally, while the conclusions about HSP90 being a potential therapeutic target are persuasive, they are based only on correlative findings and molecular docking data. To further elucidate the mechanism of action related to HSP90, CRISPR knockout technology will be utilized in animal models through gene-editing techniques.

## 5. Conclusions

Our study demonstrates that TP administration mitigates the release of ROS, diminishes oxidative stress, and modulates the Nrf2/HO-1/GPX4 signaling pathway, thereby alleviating radiation-induced ferroptosis. Additionally, through 16S rDNA analysis of the gut microbiome and untargeted metabolomics using UHPLC-MS, we observed that TP influences the composition, diversity, and stability of the gut microbiota. These findings offer important insights that could guide future studies on utilizing TCM as a potential therapeutic approach for mitigating radiation-induced harm.

## Figures and Tables

**Table 1:** The identification of 8 components in TP by UPLC-Q/TOF-MS.

No.	t_R_ (min)	MS ^1^ (*m*/*z*)	Formula	Error (ppm)	MS ^2^ (*m*/*z*)	Compounds
1	1.33	305.06652 [M-H]^−^	C_15_H_14_O_7_	−2.3	109,125,137,169,177,289	Epigallocatehin
2	0.65	305.06597 [M-H]^−^	C_15_H_14_O_7_	−0.5	125,269	Gallocatehin
3	2.13	289.07148 [M-H]^−^	C_15_H_14_O_6_	−1	124,125,137,271	Cianidanol
4	0.85	289.07088 [M-H]^−^	C_15_H14O_6_	−3	109,123,151	Epicatehin
5	2.7	273.07625 [M-H]^−^	C_15_H_14_O_5_	−2.2	125,137,255	Epiafzelechin
6	1.33	457.07745 [M-H]^−^	C_22_H_18_O_11_	−3.2	125,169,305,331,413	Epigallocatehin gallate
7	0.81	441.08234 [M-H]^−^	C_22_H_18_O_10_	−0.9	124,125,145,169,289	Catechin gallate
8	0.69	441.08222 [M-H]^−^	C_22_H_18_O_10_	−1.1	169,287	Epicatehin gallate

^1^: Primary mass spectrometry; ^2^: Secondary Mass Spectrometry.

## Data Availability

All of the data are contained within the article.

## Supplementary Materials

The following supporting information can be downloaded at: https://www.mdpi.com/article/10.3390/antiox14050580/s1, Figure S1: UPLC-Q-TOF/MS conditions for ingredient identification in TP; Figure S2:The standard curve of inflammatory factor; Figure S3: Spleen IL-6 levels; Table S1: The key active ingredients of TP in the treatment of RII; Table S2 The RNAi base sequences.

## Abbreviations

Acronyms	Definition	Acronyms	Definition
RIII	Radiation-induced intestinal injury	HO-1	Heme oxygenase 1
TP	Tea polyphenols	HE	Hematoxylin–eosin
BLI	Biolayer interference	IHC	Immunohistochemistry
HSP90	Heat Shock Protein 90	UPLC-Q-TOF/MS	Ultra-performance liquid chromatography quadrupole-time-flight mass spectrometry
GPX4	Glutathione peroxidase 4	PCoA	Principal Coordinates Analysis
IR	Ionizing radiation	PCA	Principal Component Analysis
ROS	Reactive oxygen species	LEfSe	Linear discriminant analysis effect size
FDA	Food and Drug Administration	PLSDA	Partial least-squares discriminant analysis
GSH	Glutathione	TNF-α	Tumor necrosis factor- α
MDA	Malondialdehyde	IL-1β	Interleukin-1
SOD	Superoxide dismutase	IL-6	Interleukin-6
LDH	Lactate dehydrogenase	TCM	Traditional Chinese Medicine
EGC	Epigallocatechin	DCFH-DA	Dichlorofluorescin diacetate
EC	Epicatech	Keap1	Kelch-like ECH-associated protein 1
ECG	Epicatechin gallate	TCM	Traditional Chinese Medicines
EGCG	Epigallocatechin gallate	KEGG	Kyoto Encyclopedia of Genes and Genomes
Nrf2	Nuclear respiratory factor 2	LDA	Linear discriminant analysis
