# Supplementary material for: Tea Polyphenols Mitigate Radiation-Induced Ferroptosis and Intestinal Injury by Targeting the Nrf2/HO-1/GPX4 Signaling Pathway
Source: Antioxidants (Basel). 2025 May 11;14(5):580. doi: 10.3390/antiox14050580 (PMC12108355; doi:10.3390/antiox14050580)
Supplement: Supplementary file 1 [file antioxidants-14-00580-s001.zip › antioxidants-3602419-supplementary.pdf]

UPLC-Q-TOF/MS conditions for ingredient identification in TP

The UPLC system (SYNAPT G2-MS, Waters Corporation) used a Waters Acquity UPLC HSS T3 column (100 mm × 2.1 mm, 1.8 μm) to separate compounds at 25 °C with a flow rate of 0.2 mL/min. The solvents used were 0.1% formic acid in water (A) and acetonitrile (B), with a gradient from 100% to 0% A over 20 minutes. A 1 μL sample was injected, and the samplers were held at 4 °C. Using the SYNAPT G2-MS with an ESI source, mass spectrometry was conducted, scanning 50–1200 m/z, with a sheath gas flow of 55 arb, a heater at 550 °C, spray voltages of ±4500 V, and collision energies of 20, 40, and 60 eV.

Western blotting

SDS-PAGE was used to separate 20 μg of total protein from each sample, which was then transferred to PVDF membranes and blocked. The blots were treated with HRP-linked secondary antibodies and developed using ECL, and imaged with a FluorChem® M system (Cell Biosciences, CA, USA). β-actin served as the control, with band densities analyzed by Image J.

Molecular docking

The chemical structure of EGCG was obtained from the PubChem database. Concurrently, the three-dimensional molecular structure of the HSP90, designated by the PDB identifier 7S9H, was sourced from the RCSB PDB repository. In the preparation phase, both water molecules and hydrogen atoms were systematically integrated into the protein models utilizing the functionalities provided by the Discovery Studio Client software. The molecular docking of EGCG onto these prepared structures was performed using the same computational tool. For the detailed analysis and visualization of the resultant molecular interactions, the Pymol software was employed, facilitating a thorough examination of the docking outcomes.

Bio-layer interference analysis

Binding affinity of HSP90 was determined with the Octet BLI Discovery12.2 (Germany), following Zhao et al.'s protocol[14]. HSP90 (RPA823Mu01, Cloud Clone Corp., Wuhan) was diluted in PBS with 0.02% Tween and biotinylated using a 10 mM solution at 25°C for 30 minutes. The protein was purified on a desalination column. EGCG was tested in a 96-well plate across concentrations from 3.125 to 100 μM. The interaction, monitored by SA Biosensors in PBST, included a 120-second association and 180-second dissociation. Kinetic parameters, including the dissociation constant (K_D), were calculated using a 1:1 binding model in Octet BLI Analysis software v12.2.

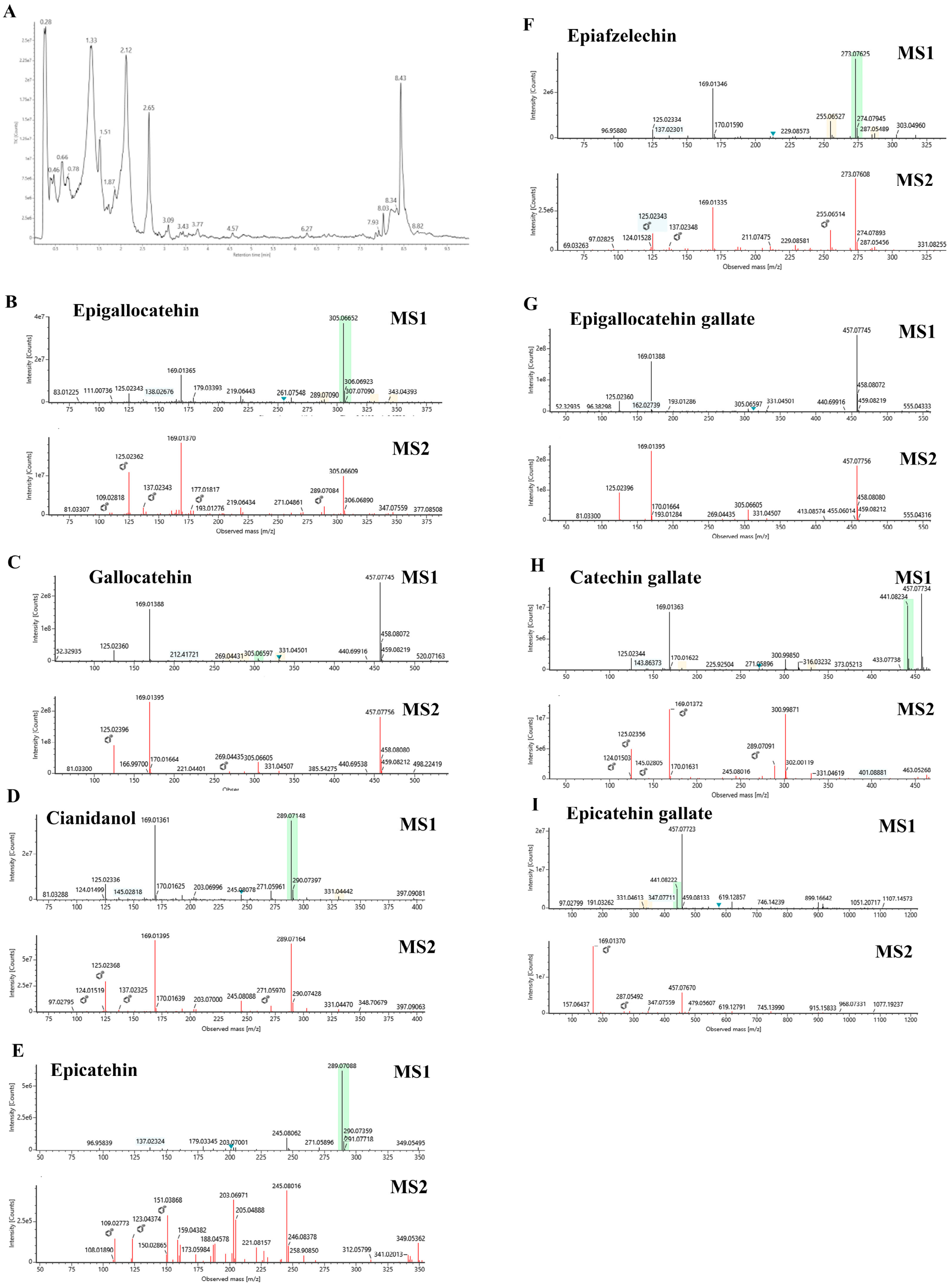

Figure S1: UPLC-Q-TOF/MS conditions for ingredient identification in TP. **A** The typical total ion chromatography (TICs) of TP. **B**-I The MS1 and MS2 of Epigallocatehin, Gallocatehin, Cianidanol, Epicatehin, Epiafzelechin, Epigallocatehin gallate, Epicatehin gallate, Catechin gallate.

Detection of inflammatory factors in mice

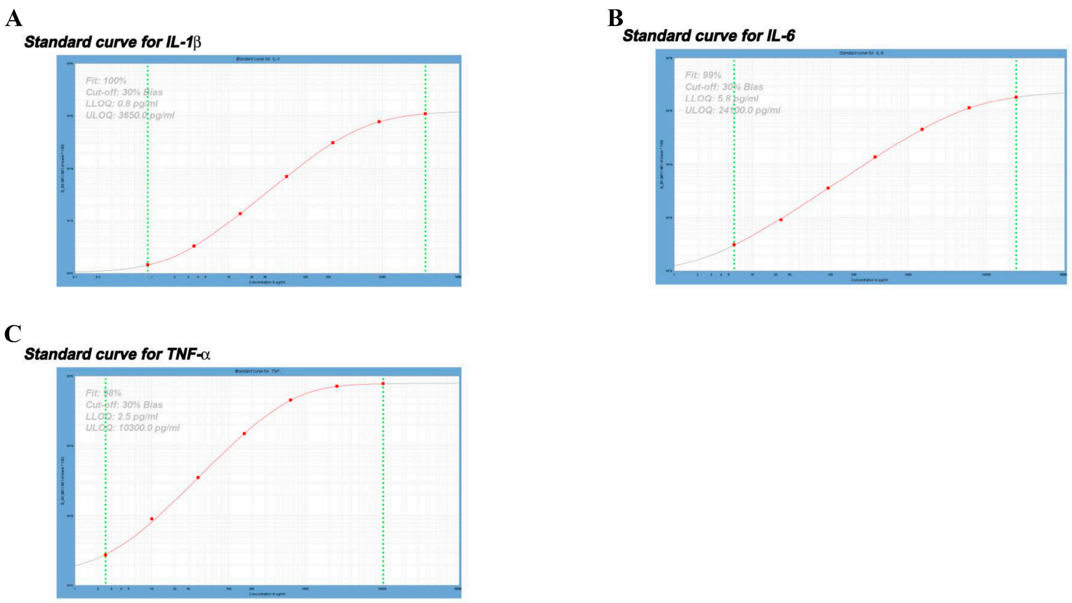

Figure S2:The standard curve of inflammatory factor. A Standar for IL-1β. B Standar for IL-6. C Standar for TNF-α

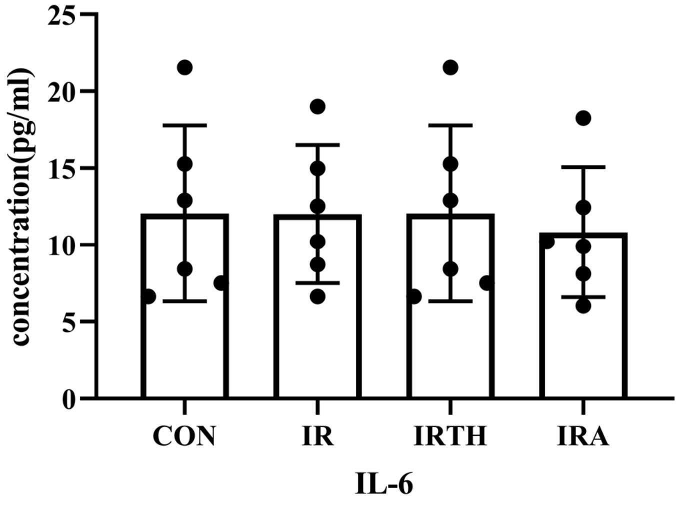

Figure S3 Spleen IL-6 levels.

Table S1 The key active ingredients of MGD in the treatment of lung cancer

No.	Compoent	SMILES
EGCG	Epigallocatechin Gallate	C1[C@H]([C@H](OC2=CC(=CC(=C21)O)O)C3=CC(=C(C(=C3)O)O)O)OC(=O)C4=CC(=C(C(=C4)O)O)O
EGC	(-)-Epigallocatechin	C1[C@H]([C@H](OC2=CC(=CC(=C21)O)O)C3=CC(=C(C(=C3)O)O)O)O
ECG	(-)-Epicatechin gallate	C1[C@H]([C@H](OC2=CC(=CC(=C21)O)O)C3=CC(=C(C=C3)O)O)OC(=O)C4=CC(=C(C(=C4)O)O)O
EC	(-)-Epicatechin	C1[C@H]([C@H](OC2=CC(=CC(=C21)O)O)C3=CC(=C(C=C3)O)O)O
GCG	Gallocatechin gallate	C1[C@@H]([C@H](OC2=CC(=CC(=C21)O)O)C3=CC(=C(C(=C3)O)O)O)OC(=O)C4=CC(=C(C(=C4)O)O)O
GC	(-)-Epigallocatechin	C1[C@H]([C@H](OC2=CC(=CC(=C21)O)O)C3=CC(=C(C(=C3)O)O)O)O
CG	catechin gallate	C1[C@@H]([C@H](OC2=CC(=CC(=C21)O)O)C3=CC(=C(C=C3)O)O)OC(=O)C4=CC(=C(C(=C4)O)O)O
CA	(+)-Catechin	C1[C@@H]([C@H](OC2=CC(=CC(=C21)O)O)C3=CC(=C(C=C3)O)O)O
GA	Gallic acid	C1=C(C=C(C(=C1O)O)O)C(=O)O

Table S2 The RNAi base sequences

No.	Base sequence
SiRNA1	CUAUCUAGUUGCAGAGAAATT<br>UUUCUCUGCAACUAGAUAGTT
SiRNA2	GGAAGAUGACAGCGGCAAATT<br>UUUGCCGCUGUCAUCUUCCTT
SiRNA3	GGAAGAGAAAGGUGAGAAATT<br>UUUCUCACCUUUCUCUUCCTT
CONTROL	CAGAGTATGTGCTCGCATGAAG<br>GGCTCAGTCATATACACCACCTC
